# Heat shock: Impact of moderate temperature on pollen development in maize

**DOI:** 10.1093/plphys/kiae207

**Published:** 2024-04-09

**Authors:** Prateek Jain

**Affiliations:** Assistant Features Editor, Plant Physiology, American Society of Plant Biologists; Department of Biology, The University of North Carolina at Chapel Hill, Chapel Hill, NC 27599-3280, USA

Global warming has caused a significant increase in the frequency and intensity of heatwaves and hot days worldwide. Exposure to high temperatures during the plant reproductive phase can lead to a dramatic decrease in seed production, largely through abnormal pollen development (Chaturvedi et al. 2021). Due to its sequenced genome and well-studied reproductive system, maize is considered an ideal crop to study the effect of heat stress (HS). Male reproductive organs like anthers and pollens are more sensitive to temperature fluctuations than female gametophytes (Tushabe et al. 2023). Therefore, it is imperative to understand the effect of moderate temperatures on pollen development.

Maize pollen grain development begins with the maturation of diploid pollen mother cells into meiocytes, which undergo 2 rounds of meiotic division to form 4 microspores (tetrad) followed by 2 rounds of mitosis, ultimately forming tricellular pollen grains. To ensure adequate pollination in times of high temperatures, it is important to understand which stages of pollen grain development are most sensitive to heat stress. The leaf collar method allows researchers to use vegetative markers to nondestructively identify pollen developmental stage in the maize inbred line B73 (Fig. 1A) (Begcy and Dresselhaus 2017). Begcy et al. (2019) used this method to identify that a short span of HS (48 hrs) during the tetrad stage of pollen development (V11 to V12) can cause decreased seed-set in maize (Begcy et al. 2019).

**Figure 1.**
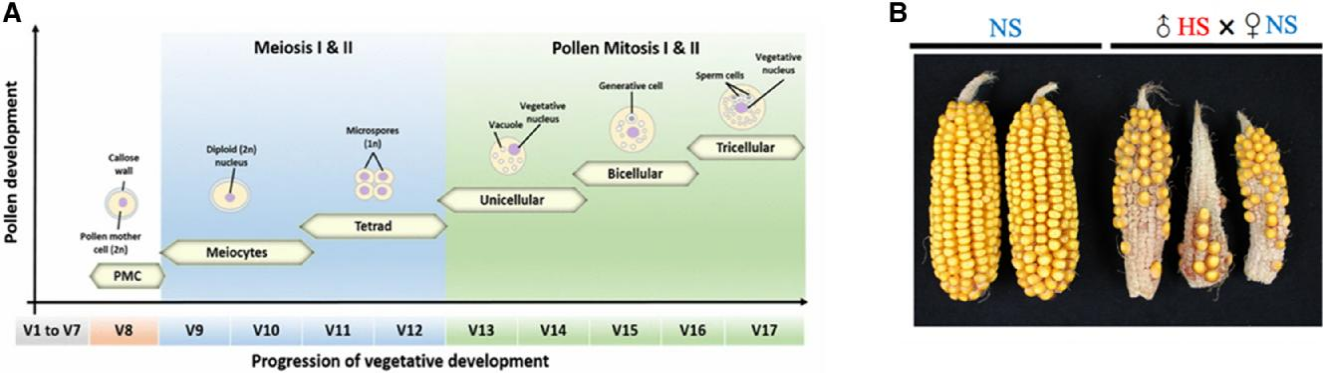
**A)** Schematic representation of progression of vegetative stages during pollen development of maize tracked by the leaf collar method (image reprinted from Begcy and Dresselhaus (2017) (Begcy and Dresselhaus 2017). **B)** Pollination of nonstressed (NS) ears with the HS pollen results in reduced seed set in maize as compared to ears fertilized by NS pollen.

In this issue of *Plant Physiology*, Li et al. (2024) investigated how moderate temperature (35 °C) affects the later stages (the unicellular and bicellular stages, V13 to V17) of pollen development (Fig. 1A) (Li et al. 2024). With the help of the leaf collar method, the authors first selected plants in their uni- and bicellular stages. The selected plants were shifted from control conditions (25°C/21°C light/dark) to moderate heat stress (35°C/25°C) for 48-hour periods at this stage of pollen development. Non–heat-stressed maize cobs pollinated with pollen from plants heat stressed at the uni- or bicelluar stage showed decreased seed-set, confirming that the detrimental effect of heat is primarily due to impacts on pollen development (Fig. 1B).

Having confirmed that heat stress is affecting the uni- and bicellular stages, the authors turned their focus to the transition from bi- to tricellular stages of pollen development. During this stage, the generative cell prepares to divide mitotically, forming the 2 sperm cells required for double fertilization to produce the embryo and endosperm. Because tubulin is a major component of the mitotic spindle, the authors examined the effects of heat on pollen development using a germ cell and sperm cell–specific α-tubulin-YFP reporter (Kliwer and Dresselhaus 2010). They observed lower expression of α-tubulin-YFP protein under HS as well as irregularities in the spindle apparatus. Furthermore, the heat-treated pollen showed deficiencies in sperm cell movement within the germinating pollen tube. The authors used liquid chromatography with tandem mass spectrometry (LC-MS) and established that the severity of sperm cell phenotype is correlated with lower tubulin levels as well as reduced levels of other cytoskeletal proteins. Hence, the study showed that moderate HS affects structural and developmental stages of pollen development.

Considering that distorted spindle assembly can lead to chromosome loss during cell division, the authors examined the impact of HS on CENH3, the centromeric histone variant, by ectopically expressing mRuby3-CENH3 in sperm cells. All 10 chromosomes of nonstressed sperm cells were labeled with mRuby3-CENH3, and although the heat-stressed sperm cells showed weaker fluorescent signals, there was no change in chromosome numbers. Finally, a transcriptome analysis from heat-stressed sperm cells revealed higher expression of cell cycle regulators like SCF complex, anaphase-promoting complex/cyclosome E-3 ligase complex, and spindle assemble checkpoint, consistent with an abnormal cell cycle in developing sperm cells.

Li et al. (2024) evaluated the effect of HS on pollen development and showed that both uni- and bicellular stages are affected by moderately elevated temperatures in maize. Furthermore, HS applied at the bicellular stage results in misregulation of cell-cycle progression genes and transport of sperm cells in the pollen tube, leading to defects in male fertility. The study provides insight into the development of climate-resilient crops, which will eventually improve yield and productivity.

## Data Availability

No new data were generated or analysed in support of this research.
